# Exploring university physical education teachers' artificial intelligence use intention profiles: a Q-methodology study

**DOI:** 10.3389/fpsyg.2026.1895365

**Published:** 2026-07-09

**Authors:** Xu Sun, Dinghua Liu, Xinyang Li, Nina Gu, Zuguo Tian

**Affiliations:** 1 School of Physical Education, Hunan University, Changsha, China; 2 School of Physical Education, Hunan Normal University, Changsha, China; 3 School of Physical Education, Hunan University of Arts and Science, Changde, China

**Keywords:** AI use intention, artificial intelligence, Q methodology, teacher subjectivity, university physical education teachers

## Abstract

**Introduction:**

Artificial intelligence is increasingly entering university teaching, research, and administrative work. This study used Q methodology to examine university physical education teachers' AI use intention profiles and to clarify how they position AI within teaching, research, and professional practice.

**Methods:**

A 42-statement Q-set was developed from theoretical models, empirical literature, policy texts, and expert interviews. Forty-five Chinese university physical education teachers completed an online Q-sorting task through Q-sortware, ranking the statements from −5 to +5 and providing explanations for their extreme choices. The Q-sorts were analyzed using Ken-Q Analysis with centroid factor extraction and varimax rotation.

**Results:**

The analysis revealed four distinct profiles with different understandings of AI use in physical education: (F1) AI as a practical assistant—improving teaching efficiency, (F2) AI within embodied boundaries—preserving professional judgment, (F3) AI for research support—enhancing academic productivity, and (F4) AI brings more risks than relief—resisting added burdens. These profiles indicate that teachers did not simply accept or reject AI, but located its value and risks differently across routine teaching support, embodied classroom judgment, academic production, and added technological burden.

**Discussion:**

These findings show that university physical education teachers' AI use intention is shaped by different professional orientations toward teaching efficiency, embodied judgment, research support, and perceived risk. Consequently, AI training and institutional support should be differentiated according to teachers' work contexts, with greater attention to the embodied, safety-sensitive, and professionally accountable nature of physical education teaching.

## 1 Introduction

Artificial intelligence (AI) is no longer merely an external tool for the digitalization of higher education. It has entered teachers' everyday professional work, including lesson preparation, classroom interaction, feedback provision, assessment design, academic writing, and administrative tasks (Crompton and Burke, 2023; Eager and Brunton, 2023). With the rapid diffusion of generative AI, teachers can use AI to generate instructional materials, design learning tasks, and support feedback and assessment. These possibilities, however, are accompanied by concerns over content accuracy, academic integrity, data privacy, and students' overreliance on AI-generated outputs (Celik et al., 2022; Chan and Hu, 2023; Moorhouse and Kohnke, 2024). The educational significance of AI, therefore, does not lie simply in its technical capacity for text generation, reasoning, or analysis, but in how teachers translate these capacities into pedagogically appropriate judgments. Recent research on teacher AI competence has similarly moved beyond technical knowledge alone, paying greater attention to beliefs, self-efficacy, professional learning, cultural conditions, and human-centered orientations (Zhou et al., 2026b). From this perspective, teachers' intention to use AI is not merely an attitude toward tool adoption, but a professional judgment shaped by efficiency demands, pedagogical improvement, professional autonomy, and risk management.

Existing research has explained teachers' AI use mainly through technology acceptance, AI competence, self-efficacy, and integration models. For instance, Scherer et al. (2019) showed that the Technology Acceptance Model and its extensions have long shaped research on teachers' technology adoption, while Chiu et al. (2025) examined teachers' confidence in using AI safely and effectively through an AI competency self-efficacy framework. More recent work has also begun to classify teachers' generative AI integration patterns, such as cautious adopters, efficiency enhancers, technology enthusiasts, and instructional innovators (Song et al., 2025). These studies have provided useful explanations of why teachers may accept, resist, or integrate AI. However, AI use intention is still often treated as a measurable variable, a competence dimension, or an integration stage. Less attention has been paid to how teachers themselves organize competing concerns over pedagogical value, workload, ethical risk, institutional expectations, and professional identity into coherent viewpoints. This limitation is particularly important in physical education, where AI use must be interpreted in relation to bodily demonstration, movement observation, classroom safety, and teachers' situated professional judgment.

The AI use intention of university physical education teachers cannot be fully explained by the general logic of teachers' technology adoption. Physical education is often enacted in dynamic settings where bodily movement, skill demonstration, peer collaboration, and safety management are closely intertwined. Teachers must continually attend to students' physical performance, exercise intensity, technical movements, and potential classroom risks (Porsanger, 2023). In this context, the value of AI in physical education cannot be reduced to efficiency gains. Rather, it depends on whether digital-intelligent technologies can be meaningfully connected with sport data collection, real-time feedback, and personalized training plans across teaching, practice, assessment, and management (Zhong et al., 2025). AI use may also support the design of teaching strategies, the tracking of learning processes, and the evaluation of learning outcomes in physical education (Wang and Wang, 2024). Yet such support does not automatically translate into professional acceptance. When AI feedback relies too heavily on quantifiable indicators, or when algorithmic judgments displace teachers' situated understanding of movement contexts, student conditions, and safety boundaries, the embodied and educative nature of physical education may be narrowed. Existing empirical research on college physical education teachers has shown a relationship between AI use intention and digital competence, but it remains necessary to explain how these teachers make sense of AI through self-efficacy, social influence, and professional adaptation (Yi et al., 2025). Thus, AI use intention among university physical education teachers is better understood as a contextualized professional judgment. It concerns not only whether AI is useful, but also whether it can be reconciled with bodily practice, classroom interaction, and teachers' professional responsibility.

Over the past 2 years, China's Ministry of Education has placed AI at the center of educational modernization through the Outline of the Plan for Building a Leading Education Power (2024–2035) and the “AI + Education” Action Plan. These policy initiatives link teachers' AI use to resource provision, teaching services, competence assessment, and educational AI agents, suggesting that AI engagement is increasingly embedded in teacher professional development, curriculum reform, and educational governance (Wang et al., 2025b). Yet policy momentum does not ensure a shared understanding among teachers. Existing studies show that teachers differ markedly in how they interpret generative AI: some emphasize its potential for curriculum innovation and instructional design (Chiu, 2024), whereas others worry that it may weaken pedagogical judgment and teacher professionalism (Prilop et al., 2025). Research on teacher subjectivity further indicates that the same educational phenomenon can be organized into different viewpoint structures, which Q methodology is well-suited to identify (Bogaers et al., 2025). In physical education, however, such evidence remains limited. Although recent qualitative work suggests that physical education teachers' understanding of AI is shaped by self-efficacy, outcome expectations, social support, and classroom suitability (Yang et al., 2026), less is known about how distinct AI use intention profiles emerge across pedagogical value, research support, and everyday professional work.

According to this research topic, the methodology must be able to capture subtle differences in teachers' viewpoints rather than reduce AI use intention to a set of predictor variables. Q methodology is well-suited to this purpose. Originating in Stephenson (1935) reworking of conventional factor analysis, it shifts the focus from correlations among variables to patterned similarities among persons' rankings of statements. Later methodological work has further positioned Q methodology as an approach for examining first-person perspectives, value judgments, and structures of meaning, particularly in topics where consensus and disagreement coexist (Watts and Stenner, 2012; McKeown and Thomas, 2013). In teacher research, it has been used to reveal reflective priorities, classroom understandings, and types of professional judgment, rather than merely estimating how many participants endorse a given view (Lim-Ratnam et al., 2022; Bogaers et al., 2025). Applied to university physical education teachers' AI use intention, Q methodology therefore allows the study to move beyond a simple distinction between supporters and opponents of AI and to identify the profiles through which teachers organize their views of AI use.

To address this research gap, this study focuses on Chinese university physical education teachers, a group situated in a distinctive professional context, and employs Q methodology to examine their subjective profiles of AI use intention. Rather than treating AI use intention as a single variable and testing its predictors, this study explores how university physical education teachers make sense of AI in their professional work. It examines whether they view AI as a tool for pedagogical innovation in physical education, an efficiency resource for research and everyday work, a potential threat to bodily demonstration, classroom interaction, and professional judgment, or a form of passive adaptation shaped by policy advocacy, institutional requirements, and peer pressure. The purpose of this study is therefore not to determine whether university physical education teachers are generally willing to use AI, but to identify how different teachers construct internally coherent AI use intention profiles around pedagogical value, work convenience, competence confidence, perceived risk, and professional identity. The study makes three contributions. Theoretically, this study moves beyond variable-centered prediction by using Q methodology to combine quantitative ranking patterns with qualitative interpretation of teachers' subjective viewpoints, thereby identifying distinct AI use intention profiles among university physical education teachers. Methodologically, it uses Q methodology to combine quantitative ranking patterns with qualitative interpretation, offering a more fine-grained approach to understanding differences within a teacher group. Practically, the findings can inform differentiated AI training, digital transformation in physical education, and institutional support mechanisms, rather than treating all teachers as targets of the same technology-promotion logic. Based on this, the study addresses the following research questions.

RQ1. What AI use intention profiles can be identified among Chinese university physical education teachers?

RQ2. How do these profiles differ in teachers' perceptions of AI use in teaching, research, and professional work?

RQ3. What professional and contextual factors may help explain the formation of these AI use intention profiles?

## 2 Literature review

### 2.1 Artificial intelligence use in university teaching practice

The integration of AI into university teaching did not begin with the recent surge of generative AI. Over the past decade, online course formats such as MOOCs, flipped classrooms, and blended learning have already brought digital technologies into the organization of university curricula (Dziubaniuk et al., 2023). What distinguishes large language models and generative AI from these earlier forms of digitalization is not simply their technical novelty, but their capacity to enter core instructional work more directly. They are now used in content generation, intelligent interaction, personalized feedback, learning design, and assessment support, thereby reshaping teaching practices, assessment arrangements, and the role of university instructors (Lee et al., 2024; O'Dea, 2024). University teachers are therefore no longer dealing only with relatively bounded systems such as learning analytics, intelligent tutoring, or automated assessment. They are also working with open-ended tools that can participate in course design, material production, feedback organization, academic writing, and everyday communication. The Digital Education Council's survey of 1,681 faculty members from 52 universities across 28 countries showed that 61% of respondents had already used AI in teaching. Among these users, 75% used AI to create teaching materials, 58% to support administrative tasks, 50% to teach students how to use and evaluate AI in class, 45% to enhance classroom engagement, 28% to detect cheating, and 24% to generate feedback on student assignments (Digital Education Council, 2025). Yet the adoption of AI in teaching is uneven across disciplines. In a systematic review of 139 studies on generative AI in higher education classrooms, Wang et al. (2025a) found that engineering, health and medicine, and language courses accounted for the most concentrated applications, whereas the humanities, social sciences, basic sciences, mathematics, physical education, and interdisciplinary fields were less represented. Some evidence further suggests that teachers in the humanities and social sciences are especially concerned that excessive dependence on AI may remove non-verbal cues from classroom interaction and weaken students' social and emotional development (Parviz, 2024). The meaning of AI in university teaching, then, cannot be assessed simply by asking whether it is used. It is necessary to examine how teachers understand different forms of AI use and how their AI use intention profiles may vary across disciplinary and professional contexts.

Assessment and feedback are among the areas in which AI has produced some of the earliest substantive changes in higher education teaching. A study of generative AI policy documents in U.S. universities shows that many institutions have moved beyond merely restricting students' AI use and have begun to provide faculty with classroom activities, syllabus statements, assessment criteria, and ethical-use guidance (Wang et al., 2024). This shift indicates that AI is being incorporated into the institutional arrangements of university teaching rather than remaining a matter of individual tool choice. Similarly, Jin et al. (2025) found that universities worldwide are developing policies and guidelines around how AI may be used and how AI involvement should be acknowledged. Such documents reshape the space within which instructors design courses and exercise pedagogical judgment. Still, institutional guidance does not mean that AI integration has become pedagogically settled. From the perspective of authentic assessment and academic integrity, Kofinas et al. (2025) showed that generative AI enables students to produce relatively high-quality written work quickly, thereby exposing the vulnerability of traditional written assignments in terms of authenticity, process evidence, and verifiability. Corbin et al. (2025) further describe the relationship between AI and higher education assessment as a wicked problem. These debates suggest that AI in university teaching cannot be reduced to efficiency improvement. Its use requires teachers to make situated judgments about different tools, tasks, and work contexts. This provides a practical foundation for examining teachers' intention to use AI in more differentiated ways.

### 2.2 Teachers' artificial intelligence use intention in professional contexts

Research on teachers' AI use intention has developed several relatively stable explanatory pathways, most commonly through variable-based approaches centered on technology acceptance, readiness, and affective experience. In the context of generative AI for teaching, Kong et al. (2024) examined Hong Kong K-12 teachers' behavioral intention to use GenAI tools through an extended technology acceptance model. Their findings suggest that teachers' intention to use AI depends not only on the tool itself, but also on how they evaluate its benefits, ease of use, others' expectations, and their own capabilities. Similarly, research on K-12 teachers' use of AI tools indicates that teachers' attitudes toward AI are not uniformly negative, although their AI self-efficacy is shaped by prior experience, perceived relevance, and school-level support (Bergdahl and Sjöberg, 2025). Evidence from teacher education further shows that preservice teachers' attitudes toward AI are significantly associated with digital self-efficacy. Those who view ChatGPT positively tend to report higher digital self-efficacy, whereas negative attitudes are often accompanied by lower technological confidence (Heine and König, 2025). Taken together, these studies show that teachers' AI use intention is not a simple matter of acceptance or rejection. It is shaped by perceived usefulness, confidence in one's capability, social influence, available support, and perceived risk. Yet this line of work also has a limitation. The AI use intention is often treated as an outcome variable in predictive models, with attention directed mainly to which factors significantly explain intention. Less attention has been paid to how teachers themselves organize these factors into professionally grounded positions with their own internal logic.

This limitation becomes more visible in teaching domains where professional context strongly shapes technology use. Teachers' AI use intention is not a psychological inclination detached from curricular tasks. It is embedded in subject knowledge, instructional organization, assessment practices, school support, and professional identity. This issue is particularly complex in physical education. Physical education involves not only knowledge transmission, but also bodily movement, athletic performance, classroom safety, and immediate feedback. Teachers' attitudes toward AI are therefore often shaped by whether they judge the technology to be capable of supporting embodied practice (Yang et al., 2026). Wu et al. (2025), in their study of AIGC acceptance among Chinese university students majoring in physical education, found that users were concerned not only with ease of use, but also with whether AIGC could genuinely support sport learning, whether sufficient environmental support was available, and whether potential risks were present. Existing research on university physical education teachers similarly indicates that AI use intention is not an isolated technological preference. It is jointly influenced by self-efficacy, outcome expectations, subjective norms, resource conditions, and teaching contexts, while remaining dynamically linked to teachers' professional competence development (Yi et al., 2025; Zhou et al., 2026a). However, research that examines university physical education teachers' AI use intention by integrating subjective meaning-making with more structured analytical evidence remains limited and requires further development.

### 2.3 Q methodology in teacher subjectivity research

The value of using Q methodology to examine university teachers' AI use intention lies in its ability to capture patterns of subjectivity that are not easily reduced to a single measurable attitude. University teachers rarely position themselves simply for or against AI. More often, they form judgments by weighing efficiency gains, pedagogical value, ethical risks, and professional identity in relation to their own teaching contexts. Brown (1996) argued, from a qualitative research perspective, that Q methodology deals with subjectivity from the standpoint of the actor. Its value is not to replace interviews or questionnaires, but to transform subjective judgments into comparable and interpretable structures. A review of Q methodology in educational research similarly shows that it has been used across topics such as teacher characteristics, learning development, student experience, and educational reform, where value conflicts and interpretive differences are often central (Lundberg et al., 2020). In teacher research, Q methodology is particularly useful for examining professional issues that appear to be shared but are understood in different ways. Chaaban et al. (2023), for example, explored teachers' career development in Qatar through 40 Q statements and the Q-sorts of 42 teachers, identifying distinct perspectives on professional development. Their study suggests that teachers' professional growth is not driven by a single factor, but shaped by the interplay of personal, social, and environmental systems. Buchholtz and Vollstedt (2024) applied Q methodology to preservice mathematics teachers' beliefs, showing that even within the same topic of mathematics teaching and learning, teachers may hold different configurations of pedagogical beliefs. These studies indicate that Q methodology can present teachers' professional concerns, value priorities, and practical understandings as profiles, rather than fragmenting teachers' perspectives into isolated variables. In higher education, Espartinez (2024) further used Q methodology to examine student and teacher perceptions of ChatGPT, showing that expectations of innovation, ethical concerns, institutional support, and cultural adaptation can coexist when AI enters university teaching. However, Q methodology research on university physical education teachers' AI use intention remains limited. What needs to be examined is not whether a single factor significantly predicts intention, but how different teachers form relatively stable AI use intention profiles across multiple dimensions, roles, and contexts.

### 2.4 Theoretical foundation

This study draws first on technology acceptance and self-efficacy perspectives to understand the basic sources of university physical education teachers' AI use intention. The Technology Acceptance Model suggests that technology use is shaped by perceived usefulness and perceived ease of use (Davis, 1989), while UTAUT further highlights performance expectancy, effort expectancy, social influence, and facilitating conditions as important determinants of use intention (Venkatesh et al., 2003). In the context of teacher AI use, these perspectives help explain why teachers may value AI for lesson planning, feedback provision, learning-material generation, or administrative workload reduction, but may hesitate when training, operational complexity, or data security remain uncertain (Shata and Hartley, 2025; Wainaina and Sun, 2025). Bandura (1997) self-efficacy theory adds another layer by emphasizing that action depends not only on external conditions, but also on individuals' judgments of their own capability. For university physical education teachers, AI self-efficacy concerns whether they believe they can transform AI tools into usable resources for teaching, research, and everyday professional work (Yang et al., 2026). Together, these perspectives informed the Q-statement domains related to AI technology acceptance, use intention, self-efficacy, and professional adaptation.

Technology acceptance and self-efficacy, however, cannot fully explain how teachers negotiate AI within professional work. University physical education teachers are not passive recipients of technological change. They act within curriculum goals, student development needs, institutional expectations, workload pressures, and ethical responsibilities. The ecological perspective on teacher agency emphasizes that teachers' actions are shaped not only by personal capacities, but also by cultural, structural, and material conditions (Priestley et al., 2026). Research on teacher beliefs similarly shows that professional action is influenced by values, educational judgments, and individual and collective discourses (Biesta et al., 2015). This perspective is especially relevant in physical education, where teaching is grounded in bodily movement, skill demonstration, immediate feedback, peer interaction, and safety management. Embodied approaches to physical education suggest that bodily experience, movement exploration, and relational learning are central to meaningful physical education (Aartun et al., 2022; Volshøj et al., 2025). When AI enters this context, teachers are likely to consider not only whether it improves efficiency, but also whether it supports motor learning, bodily experience, movement safety, and educative purposes. In this sense, AI adoption in physical education cannot be understood only as a general technology acceptance issue. It also involves teachers' situated judgments about the boundary between algorithmic support and embodied professional practice. This subject-specific complexity makes a subjective, profile-based approach necessary, because teachers may organize concerns about efficiency, bodily demonstration, movement feedback, safety responsibility, and professional identity in different ways. These perspectives, therefore, informed the Q-statement domains related to AI-enabled physical education practice, risk perception, and professional identity.

This study does not treat TAM, UTAUT, self-efficacy theory, teacher agency, and embodied physical education as separate hypothesis-testing models. Instead, they serve as a sensitizing framework for developing the concourse, constructing the Q-set, and interpreting the factor structures. Their role is to ensure that the statement pool covers the main ways in which university physical education teachers may understand AI use intention, including perceived usefulness, capability beliefs, institutional conditions, embodied teaching practice, and professional risk. In this sense, theory does not predetermine the results of the Q analysis. Rather, it supports a broad and context-sensitive basis for identifying and interpreting the AI use intention profiles that emerge from teachers' own sorting patterns.

## 3 Methodology

### 3.1 Research design

This study employed Q methodology to examine the AI use intention profiles of Chinese university physical education teachers. AI use among university physical education teachers is likely to vary across individuals. Some teachers may value AI for its efficiency in lesson preparation, research writing, and classroom feedback, whereas others may be more concerned about its potential influence on bodily demonstration, classroom interaction, and judgments about sport safety. Still others may adopt a cautious adaptive stance shaped by institutional policy advocacy, peer pressure, and their own technological confidence. Q methodology is well suited to this purpose because it shifts the analytical focus from correlations among variables to correlations among individual rankings, thereby identifying shared structures of subjective viewpoints through participants' sorting of statements (McKeown and Thomas, 2013). Originating in Stephenson (1935) reworking of conventional factor analysis, Q methodology provides a systematic way to study subjectivity by examining how participants organize their viewpoints around a given topic. Watts and Stenner (2012) further argue that a well-designed Q study can reveal the key viewpoints within a participant group and interpret them holistically with rich qualitative detail. Unlike conventional Likert-scale approaches, Q methodology uses by-person factor analysis to examine similarities among participants' Q-sorts rather than relationships among variables; it can therefore capture consensus, difference, and distinctive viewpoints within the same analysis (Lundberg et al., 2020). In teacher research, Chaaban et al. (2023) showed that Q-sorting and factor interpretation can identify different viewpoint types in teachers' career development, rather than merely quantifying teachers' agreement with separate factors. Accordingly, following the methodological logic of Q and drawing on the implementation guidance of Watts and Stenner (2012), this study proceeded through five interrelated stages: Q-set development, participant selection, Q-sorting, Q factor analysis, and factor interpretation. These stages were used to identify the AI use intention profiles of Chinese university physical education teachers.

### 3.2 Q-set development

Q-set development connected the theoretical framework of this study with the empirical sorting task. Brown et al. (2020) noted that moving from a concourse to a Q sample involves transforming narratives and viewpoints into sortable statements, rather than merely reducing the amount of material. Following this logic, an initial concourse was developed around Chinese university physical education teachers' AI use intention. Two types of sources were used. Informational sources included literature on technology acceptance, self-efficacy, teacher professional agency, and embodied physical education, as well as studies on teacher AI use, AI applications in physical education, and relevant Chinese policy documents. Conversational sources were drawn from interviews with five experts in physical education. These interviews focused on typical AI-use scenarios, perceived benefits, practical concerns, pedagogical fit, and possible implications for teachers' professional identity. The interview data were not copied directly into the final Q-set. Instead, they were used to enrich the more context-specific expressions that were less visible in published literature and policy texts. The initial statement pool was developed with the support of two physical education professors and one English professor, who reviewed whether the statements were clear, concise, and appropriate for the context of university physical education.

After the concourse had been developed, the research team organized 90 candidate statements around five discourse domains: AI technology acceptance and use intention, AI self-efficacy and professional adaptation, AI-enabled physical education practice, AI-supported research and daily work, and AI risk perception and professional identity. Statements were removed or rewritten when they were semantically repetitive, ambiguous, double-barreled, overly abstract, or difficult to sort. Five experts in physical education reviewed the revised pool, focusing on relevance, clarity, coverage, and sortability. On the basis of their feedback, the research team merged similar statements, adjusted the strength of several statements, and sought to maintain a reasonable balance across the five discourse domains. A pilot test was then conducted with 10 university physical education teachers to check whether the statements were understandable, sufficiently distinguishable, and manageable within the sorting task. The pilot data were used only for refining the Q-set and were not included in the final analysis. After expert review and pilot testing, the final Q-set consisted of 42 statements. The complete Q-set is provided in Appendix 1.

### 3.3 Participants and Q-set construction

Forty-five Chinese university physical education teachers were recruited through purposive sampling to form the P-set. They were recruited from 10 Chinese universities, including five sports universities and five normal universities. This sampling strategy was intended to enhance institutional diversity and broaden the range of viewpoints represented in the P-set. In Q methodology, participant selection is not intended to achieve statistical representativeness, rather, it aims to include participants who are sufficiently familiar with the topic and able to offer relevant and diverse viewpoints. Therefore, the P-set in this study should not be understood as a statistically representative sample of university physical education teachers across all regions of China. Its purpose was to capture information-rich and varied viewpoints on AI use intention among university physical education teachers. Watts and Stenner (2012) noted that Q studies do not require large numbers of participants, but need enough participants to establish the existence of factors. Similarly, Webler et al. (2009) indicated that Q studies commonly work with several dozen Q-sorts, which can be analyzed through correlation and factor analysis to reveal patterns of shared perspectives. On this methodological basis, 45 participants were considered appropriate for identifying shared AI use intention viewpoints within the present Q-sample.

All participants had experience teaching physical education in Chinese universities and had basic knowledge of, or prior exposure to, AI use in teaching, research, or everyday professional work. To increase viewpoint diversity, recruitment considered gender, teaching experience, and academic title. Participants' demographic and professional characteristics are presented in Table 1. The study was approved by the Ethics Committee of Hunan Normal University (approval number: 2026391). Before completing the Q-sort, all participants read the study information sheet and provided informed consent. The study followed the Declaration of Helsinki. Participants were informed that participation was voluntary, that they could withdraw at any time, and that all data would be used only for academic purposes and stored and analyzed anonymously.

**Table 1:** Participant characteristics of the P-set.

Variables	Description	*N*	%
Gender	Male	23	51.1
	Female	22	48.9
Academic title	Lecturer	23	51.1
	Associate professor	13	28.9
	Professor	9	20.0
Teaching experience	5 years or less	8	17.8
	6–10 years	16	35.6
	11–20 years	15	33.3
	More than 20 years	6	13.3

**Figure 1:**
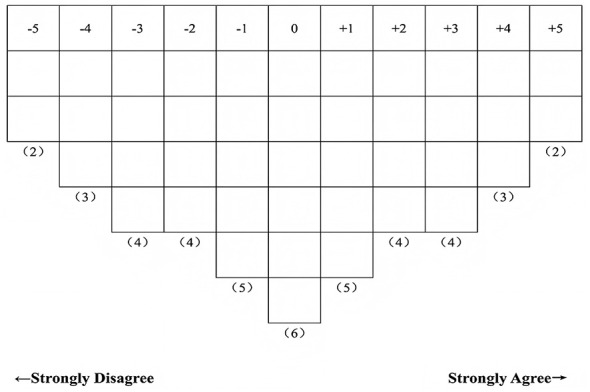
Q-sort distribution grid.

Q-sorting was conducted online using Q-sortware. The platform is specifically designed for online data collection in Q methodology, while broader Q methodology software platforms are commonly used to build Q studies, collect Q-sorts, and export or analyze study data. After entering the system, participants first read the condition of instruction and sorted each statement according to the extent to which it reflected their view of AI use in university physical education teaching, research, and professional work. They were asked to place the 42 statements into three preliminary piles: agree, neutral, and disagree. They then arranged the statements on a forced quasi-normal distribution grid ranging from −5 to +5 (Figure 1). A score of −5 indicated that a statement was least consistent with the participant's view, +5 indicated that it was most consistent, and 0 indicated a relatively neutral or uncertain position. During the sorting process, participants compared statements in relation to one another rather than judging each statement in isolation. After completing the sort, they provided written explanations for the statements placed at the extreme positions, particularly +5, +4, −4, and −5. These post-sort comments were collected to support later factor interpretation, so that the resulting AI use intention profiles would not be explained solely from numerical rankings but also from participants' own accounts of their choices.

### 3.4 Q-sorting procedure and factor interpretation

The Q-sorts completed by the 45 university physical education teachers were imported into Ken-Q Analysis for data analysis. Ken-Q Analysis is a web-based tool for Q methodology that supports the calculation of Q-sort correlations, factor extraction, factor rotation, and the generation of factor arrays, making it suitable for transforming participants' sorting patterns into interpretable viewpoint structures. In this study, by-person factor analysis was used, with each participant's Q-sort treated as the unit of analysis. Centroid factor analysis was applied for factor extraction, followed by varimax rotation to obtain a clearer factor structure. These procedures are consistent with common Q-methodological practice, where centroid or principal component extraction is typically followed by factor rotation, often using varimax or manual rotation. The final factor solution was not determined by software output alone. Eigenvalues, explained variance, significant factor loadings, the number of defining Q-sorts for each factor, factor interpretability, and the theoretical relevance of the research question were considered together. Specifically, we examined three-, four-, and five-factor solutions. The three-factor solution was not selected because it risked under-extraction. It compressed substantively different viewpoints into broader factors and would have left out one factor with an eigenvalue greater than 1, thereby reducing the breadth of the viewpoint structure captured in the analysis. The five-factor solution was also rejected because the fifth factor had an eigenvalue of 0.9688, which was below the eigenvalue-greater-than-one criterion. Although this value was close to 1, the fifth factor was defined by only one Q-sort after varimax rotation, making it difficult to justify as a stable and shared viewpoint. Compared with these alternatives, the four-factor solution provided the most defensible balance among statistical adequacy, defining Q-sorts, interpretability, theoretical relevance, and parsimony.

Significant factor loadings were calculated using Brown and Stephenson (1980) formula, 2.58 × (1 ÷ √number of statements in the Q-set), to determine whether a Q-sort loaded significantly on a factor at the *p* < 0.01 level. The Q-set contained 42 statements; therefore, the threshold for a significant loading was approximately ±0.40. As shown in Table 2, all 45 Q-sorts reached a significant loading. However, three Q-sorts (T03, T28, and T44) loaded significantly on two or more factors and were therefore treated as confounded cases. They were excluded not only from the construction of the final factor arrays but also from factor-specific interpretation and factor naming. Their post-sort explanations were not used as primary evidence for any single profile, in order to avoid assigning ambiguous viewpoint structures to any single factor and to preserve interpretive clarity. The retained solution consisted of four factors: Factor 1 with 15 defining sorts and 36% explained variance, Factor 2 with 13 defining sorts and 28% explained variance, Factor 3 with 7 defining sorts and 10% explained variance, and Factor 4 with 7 defining sorts and 7% explained variance.

**Table 2:** Quantitative summary of the four-factor solution.

Factor	F-1	F-2	F-3	F-4	Confounded cases
Number of defining Q-sorts (participants)	15	13	7	7	3
Explained variance	36%	28%	10%	7%	–

Factor interpretation was based on the statistical results, but it was not limited to reading the software output. In Q methodology, each factor represents a shared viewpoint structure. The factor array is the idealized Q-sort generated from the Q-sorts that load significantly on the same factor, and it provides the basis for interpreting the meaning of that factor. Watts and Stenner (2012) emphasize that Q factor interpretation should attend to both statistical patterns and participants' qualitative explanations of their sorting decisions. Accordingly, this study interpreted and named the factors by examining several sources of evidence: the factor arrays, the highest- and lowest-ranked statements, statements ranked notably higher or lower in one factor than in others, distinguishing statements, consensus statements, and participants' post-sort explanations for the statements placed at +5, +4, −4, and −5. The factor labels were not assigned mechanically from extreme statements alone. Instead, they were developed through repeated comparison of the factor arrays, cross-factor differences, and participants' written explanations. Through this process, the statistical structures identified in the Q-sorting data were translated into theoretically meaningful and practically relevant AI use intention profiles among university physical education teachers.

## 4 Results

A four-factor solution (F-1 to F-4) was extracted, representing four distinct viewpoint structures regarding Chinese university physical education teachers' intention to use AI. The results are presented by integrating the quantitative factor arrays with participants' post-sorting explanations, so that each profile is interpreted through the overall configuration of statements rather than isolated high- or low-ranked items. In reporting the results, the statement number and its ranking in a given factor are shown in parentheses. For example, (8; 4) indicates that Statement 8 was ranked at +4 in that factor. Participant codes in the excerpts follow the same anonymized format, with T01 referring to Teacher 01. Based on the extreme-ranked statements, distinguishing statements, consensus statements, and participants' supplementary comments, the four factors were labeled as follows. F-1: AI as a practical assistant—improving teaching efficiency, F-2: AI within embodied boundaries—preserving professional judgment, F-3: AI for research support—enhancing academic productivity, and F-4: AI brings more risk than relief—resisting added burdens.

### 4.1 Factor 1: AI as a practical assistant—improving teaching efficiency

**Figure 2:**
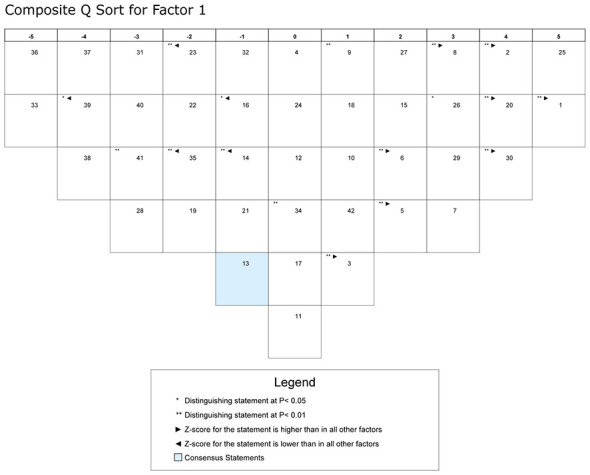
Factor array F-1.

Fifteen participants loaded significantly on F-1, including seven males and eight females. F-1 accounted for 36% of the variance, representing the largest share of explained variance within this Q-sample (Figure 2). These teachers strongly agreed that AI is better positioned as a supportive tool in physical education teaching (25; 5), and that it can improve the efficiency of their teaching work (1; 5). At the same time, they did not believe that AI would impose additional workload on teachers (36; −5), nor did they view AI use as a source of substantial academic integrity pressure (33; −5). Their intention to use AI was therefore mainly grounded in its perceived contribution to work efficiency.

In specific work settings, these teachers preferred AI tools that are easy to operate (2; 4). They also believed that AI could help organize and analyze physical fitness test data (20; 4), and support some administrative writing tasks (30; 4). These functions were closely related to the routine workload of university physical education teachers. By contrast, they did not believe that AI would substantially increase technical work requirements (37; −4), nor did they regard information overload as a prominent problem in AI use (39; −4). Excerpt 1 below presents the views of a university physical education teacher (T01) on the use of AI.

Excerpt 1.

I believe AI can support physical education teachers by helping us process physical fitness test data more quickly, conduct preliminary analysis, and free up time for classroom teaching and student guidance. (T01).

In classroom teaching, these teachers did not show an unreservedly expansive view of AI. They agreed that AI could support the processing of instructional information (8; 3), and they also held a moderately positive view of AI-assisted research or project material preparation (29; 3). However, they did not express clear confidence in AI's capacity to handle the complex situations of physical education classes (24; 0). Their endorsement of AI for differentiated exercise recommendations (5; 2) and personalized teaching arrangements (6; 2) also remained moderate. This pattern suggests that they were willing to use AI in specific, controllable, and relatively low-risk tasks.

Consistent with this pragmatic orientation, these teachers acknowledged that responsibility issues may arise in AI use (42; 1). However, they clearly rejected the view that AI would weaken teachers' professional autonomy or space for on-site judgment (38; −4). They also did not treat assessment fairness (41; −3) or privacy risks (40; −3) as major concerns. Meanwhile, they were not inclined to prioritize AI use in research work (31; −3), and their endorsement of AI-assisted research design was low (28; −3). They also did not believe that motor skill instruction should primarily rely on AI for on-site judgment (23; −2), nor did they regard AI as an important tool for carrying the value of teachers' physical demonstration (35; −2). Overall, F-1 teachers' intention to use AI was mainly confined to supportive, instrumental, and routine work-related functions.

### 4.2 Factor 2: AI within embodied boundaries—preserving professional judgment

**Figure 3:**
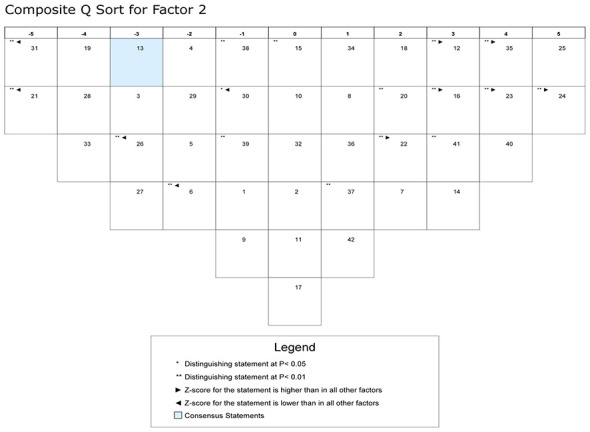
Factor array F-2.

Thirteen participants loaded significantly on F-2, including eight males and five females. This factor explained 28% of the variance (Figure 3). Similar to F-1, these teachers strongly agreed that AI is better positioned as a supportive tool in physical education teaching (25; 5). However, they also believed that the complexity of physical education classes would limit the practical effectiveness of AI applications (24; 5). This boundary-oriented view was especially evident in their understanding of the embodied nature of physical education. They strongly agreed that teachers' physical demonstrations have unique value (35; 4), and that motor skill instruction relies heavily on teachers' on-site judgment (23; 4). At the same time, they were not inclined to prioritize AI use in research work (31; −5), nor did they believe that AI could help teachers promptly understand changes in students' athletic performance (21; −5). These rankings suggest that F-2 teachers did not reject the practical value of AI, but insisted that its use should remain within the scope of teachers' professional judgment in physical education. This view is reflected in T14′s explanation in Excerpt 2.

Excerpt 2.

AI can be used for post-class analysis, but movement correction, skill demonstration, and classroom judgment in physical education still depend on teachers' on-site judgment. (T14)

In classroom teaching, these teachers believed they were able to judge whether AI tools were suitable for physical education contexts (12; 3). They also recognized that teachers' adaptation to AI depends on their professional judgment of the context of use (16; 3), and emphasized the need to identify problems in AI-generated content (14; 3). Even so, they did not show a strong willingness to actively explore AI applications in teaching (3; −3), nor did they believe they could effectively transform AI-generated content into physical education teaching materials (13; −3). Their concern was therefore not simply whether AI could be used, but whether it fitted specific teaching tasks without crossing the boundaries of teachers' professional judgment. They showed limited agreement that AI could improve the efficiency of organizing students' physical fitness test data (20; 2), and provide differentiated exercise recommendations for students (18; 2). By contrast, they did not believe that AI could effectively assist in analyzing students' movement techniques (19; −4), and they expressed no positive view of AI-designed personalized physical education plans (17; 0).

Their attitude toward AI in research and academic work was even more reserved. They did not believe that AI could effectively support research design (28; −4), and they were skeptical about its role in improving English academic writing (27; −3). AI-assisted literature searching was also not positively endorsed (26; −3), and AI support for grant proposal writing was ranked negatively (29; −2). Nevertheless, these teachers did not overstate the general technological risks of AI. They did not believe that learning AI would substantially increase their workload (38; −1), nor did they regard information overload as a particularly salient pressure (39; −1). Their concerns were more closely tied to professional and ethical issues in physical education. They tended to agree that AI-based evaluation of students' athletic performance may raise fairness concerns (41; 3), and believed that processing students' athletic data with AI could involve privacy risks (40; 4). Overall, F-2 teachers accepted AI as a supportive tool in physical education, but preferred to use it within a professionally controllable range rather than allowing it to lead motor skill instruction, student evaluation, or academic research.

### 4.3 Factor 3: AI for research support—enhancing academic productivity

**Figure 4:**
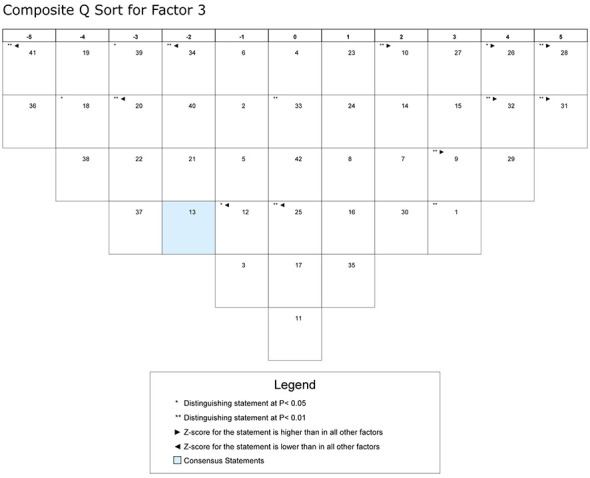
Factor array F-3.

Seven participants loaded significantly on F-3, including four males and three females. This factor explained 10% of the variance (Figure 4). These teachers did not locate their intention to use AI primarily in its role as a supportive tool for classroom teaching (25; 0). Instead, they strongly agreed that AI could support research design (28; 5), and they showed a clear preference for using AI first in research work (31; 5). By contrast, they strongly disagreed that AI-based evaluation of students' athletic performance would raise fairness concerns (41; −5), and they did not believe that AI-based evaluation would weaken physical education teachers' professional authority (36; −5). This pattern suggests that their intention to use AI was oriented more toward research activity and knowledge production than toward classroom evaluation.

When AI was considered in relation to research tasks, these teachers emphasized its role in supporting the academic production process. They believed that AI could improve the efficiency of literature searching (26; 4), and that it could assist with the preparation of grant application materials (29; 4). This positive orientation, however, did not amount to unconditional reliance on AI-generated content. They also stressed that teachers' research judgment should guide the use of AI-generated outputs (32; 4). In this factor, AI was therefore positioned as a research support tool rather than a substitute for teachers' theoretical choices, variable selection, or academic argumentation. These teachers also did not believe that learning to use AI would add substantially to their workload (38; −4), nor did they regard information overload as a major barrier (39; −3). Taken together, these rankings indicate a strong sense of task fit between AI use and the growing research demands faced by Chinese university physical education teachers. Excerpt 3 illustrates one participant's experience of using AI in research.

Excerpt 3.

AI can help me quickly expand keywords, map relationships between research topics, and notice research directions that are easy to overlook. (T32)

When the focus shifted to teaching-related applications, these teachers were less positive about AI. They did not believe that AI could effectively assist in analyzing students' movement techniques (19; −4), nor did they endorse its capacity to provide differentiated exercise recommendations (18; −4). Even physical fitness test data processing, a relatively digitized task, was not positively ranked (20; −3). AI support for theoretical physical education classes was also not clearly endorsed (22; −3). These rankings indicate that their AI use was concentrated in academic work, including research design, literature processing, project materials, and manuscript preparation.

To some extent, they regarded AI competence as part of professional development (10; 2), and they acknowledged the need to identify problems in AI-generated content (14; 2). They also showed some agreement that AI could improve the efficiency of physical education teaching (1; 3), although teaching efficiency was not the central concern of this factor. At the same time, they rejected the view that AI would increase technical work requirements (37; −3), and they did not show strong concern about privacy risks in student athletic data (40; −2). Overall, F-3 teachers' intention to use AI was centered on research tasks and remained premised on teachers' own professional judgment.

### 4.4 Factor 4: AI brings more risks than relief—resisting added burdens

**Figure 5:**
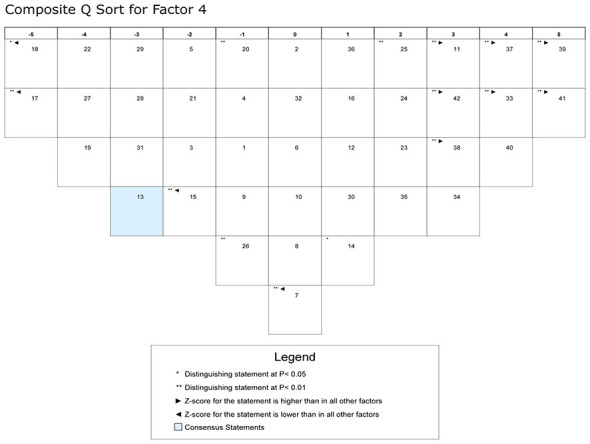
Factor array F-4.

Seven participants loaded significantly on F-4, including two males and five females. This factor explained 7% of the variance (Figure 5). For these teachers, AI use was associated more with risk and burden than with clear professional benefit. They strongly believed that the proliferation of AI tools would lead to information overload (39; 5), and that AI-based evaluation of students' athletic performance could raise fairness concerns (41; 5). Although they did not completely dismiss AI as a supportive tool in physical education teaching (25; 2), this limited recognition did not extend to core instructional functions. They did not believe that AI could provide differentiated exercise recommendations for students (18; −5), nor did they believe that AI could design personalized physical education programs (17; −5). They also rejected the view that AI could assist motor skill instruction (19; −4), and showed a clearly negative view of AI use in theoretical physical education classes (22; −4). This concern was also reflected in T40′s explanation in Excerpt 4.

Excerpt 4.

There are now too many AI tools. Physical education teachers have to judge and select suitable tools, and this itself creates a new burden. (T40)

This risk-oriented view was also evident in their perceptions of workload and responsibility boundaries. These teachers believed that AI would increase the technical demands placed on physical education teachers (37; 4), and that AI use would bring greater pressure related to academic standards (33; 4). They also believed that wider AI use could create privacy risks in the processing of students' athletic data (40; 4), and regarded responsibility boundaries in AI use as an issue requiring attention (42; 3). Consistent with this view, they also agreed that learning to use AI could add to teachers' workload (38; 3). For F-4 teachers, AI was therefore not primarily understood as a tool for efficiency improvement, but as a source of added technical, ethical, and professional demands.

Compared with other participants, these teachers did not view AI as a stable resource for improving physical education teaching. They showed mild disagreement with the claim that AI could improve the efficiency of physical education teaching (1; −1), and they did not positively endorse its use in organizing and analyzing students' physical fitness test data (20; −1). They acknowledged the value of teachers' physical demonstration to some extent (35; 2), and also indicated that AI use in motor skill instruction must take account of the complexity of physical education classes and remain dependent on teachers' on-site judgment (23; 2). These rankings suggest that F-4 teachers were more inclined to preserve the situated and embodied character of physical education teaching than to expand AI-based instructional support.

In research and professional development, these teachers also showed limited intention to use AI. They did not believe that AI could effectively support research design (28; −3), nor were they inclined to prioritize AI use in research work (31; −3). They also did not positively endorse AI support for grant application materials, a common task for university teachers (29; −3), and held a clearly negative view of AI's role in improving English academic writing (27; −4). By comparison, they showed only mild agreement that teachers need to identify problems in AI-generated content (14; 1), and only limited acceptance of AI-assisted administrative writing (30; 1). Overall, F-4 teachers approached AI use with caution, as they perceived its risks and added burdens to outweigh its practical benefits.

## 5 Discussion

The four profiles indicate that AI use intention among Chinese university physical education teachers cannot be placed on a single continuum from acceptance to resistance. Rather, it emerges from teachers' judgments about how AI fits different parts of their professional work. This study extends recent reviews showing that educators' AI adoption is shaped not only by individual beliefs and infrastructure, but also by tool compatibility, ethical concerns, transparency, and perceived effects on professional work (Taheri et al., 2025). It also resonates with emerging research on AI in physical education, where AI is seen as promising for personalization, feedback, and performance analysis, yet its pedagogical use remains underexplored and context-sensitive (Bofill et al., 2025). Thus, the following discussion treats AI use intention as a situated professional response shaped by work roles, embodied pedagogy, and contextual risk.

The efficiency-oriented profile accounted for the largest share of explained variance within this Q-sample, but this should not be read as evidence of broad technological optimism among university physical education teachers. It shows instead that AI became acceptable when attached to repetitive, data-based, and text-based work. This task-specific acceptance is understandable in the Chinese university context, where teaching, research, and administrative duties jointly shape teachers' workload and stress (Xu et al., 2023). In physical education, studies of teachers' technology practices show that technology use is shaped by task relevance, informal learning, peer support, and practical teaching constraints rather than by abstract enthusiasm for innovation (Baek et al., 2018; Ha et al., 2024). The present study adds that, before AI is trusted as a tool for transforming physical education teaching, it may first gain acceptance through low-risk and verifiable tasks that reduce routine work. The contrast between F-1 and F-4 is therefore central. For F-1 teachers, AI compressed the visible repetitive work. For F-4 teachers, the same technological environment created new digital labor, including tool selection, output verification, data interpretation, and responsibility explanation. This tension is consistent with physical education-specific discussions of technostress, where digital platforms, learning management systems, fitness software, and technology-mediated resources may increase teachers' workload and uncertainty when institutional support is insufficient (Lee et al., 2025). F-1 therefore reflects a task-specific intention to use AI as a controllable efficiency tool, valued for reducing existing work without creating additional digital labor.

The embodied-boundary profile shows that AI use intention was filtered through the activity-based nature of physical education teaching. Physical education involves more than knowledge transmission. It includes embodied practices such as physical demonstration, movement observation, immediate correction, exercise load regulation, classroom organization, and safety management (Volshøj et al., 2025). Technology use in physical education, therefore, needs to be embedded in concrete movement contexts and instructional tasks, rather than judged only by the tool's functional value (Sullivan et al., 2024). This helps explain why F-2 teachers insisted that AI should remain subordinate to teachers' on-site judgment and bodily demonstration. This boundary was not limited to F-2. F-1 teachers accepted AI for physical fitness data, teaching information, and routine materials, but did not extend this acceptance to on-site judgment in motor skill instruction. F-4 teachers were more skeptical about AI for movement analysis, differentiated exercise suggestions, and personalized physical education plans. Although digital-intelligent technologies are increasingly discussed in relation to motion monitoring, real-time feedback, learning analytics, and personalized support in physical education (Zhong et al., 2025), these functions do not automatically generate pedagogical trust. Statement 13, the blue-marked consensus statement in Figures 2–5, captures this translation problem: across the four profiles, teachers did not strongly agree that AI-generated content could be transformed into physical education teaching materials. The issue, then, lies less in AI's capacity to generate content than in the pedagogical work required to reshape its outputs into teachable, accountable, and context-appropriate physical education materials.

The research-support profile shows that AI use intention was shaped not only by classroom needs, but also by research evaluation, grant applications, and journal publication. Much of the existing literature on teachers' AI use has framed adoption through classroom technology, focusing on teaching resource generation, learning feedback, personalized support, and classroom decision-making (Tan et al., 2025). Such a classroom-centered perspective does not fully capture the dual role of university physical education teachers as educators and researchers. Research on Chinese higher education-based physical education teacher educators has shown that involvement in research activities is an important professional learning need, particularly in relation to formulating research questions, selecting research methods, and communicating research findings (Gong et al., 2024). In the wider Chinese university context, pressure to publish in internationally indexed journals and meet promotion-related research requirements has also been well documented, especially among early-career academics (Tian et al., 2016). Against this background, AI becomes attractive not simply because it can assist teaching, but because it can support idea generation, literature synthesis, content structuring, language editing, and research workflow management (Khalifa and Albadawy, 2024). Still, F-3 does not suggest that AI replaces teachers' academic judgment. Studies of generative AI in research similarly stress that researchers must retain control over problem formulation, evidence verification, originality, and academic integrity (Andersen et al., 2025; Salman et al., 2025). Read alongside F-1, F-3 broadens the meaning of efficiency from routine work reduction to academic productivity. AI use intention among university physical education teachers should therefore be understood not only as classroom technology adoption, but also as professional work adoption across teaching, research, project writing, and administrative tasks.

The risk-burden profile offers a necessary caution for AI implementation in physical education. F-4 suggests that university physical education teachers' reservations about AI are not simply individual negativity, but contextual responses to information overload, privacy risks, assessment fairness, unclear responsibility boundaries, and normative pressure that may accompany institutionalized AI use (Chinoracky and Stalmasekova, 2025). Research in physical education has highlighted the potential of AI and digital technologies for motion tracking, real-time feedback, performance analysis, and personalized support (Wang and Wang, 2024; Martín-Rodríguez and Madrigal-Cerezo, 2025). Yet when such technologies are used to collect students' movement data, assess athletic performance, or manage classroom activity, they also raise questions about data ethics, algorithmic transparency, teacher accountability, and students' bodily rights. This concern is supported by sport-related AI ethics research, which identifies privacy, fairness and bias, transparency and explainability, and accountability as central issues in AI applications (Kim et al., 2025). In this sense, F-4 should not be read as a rejection of AI itself. It signals the need to prevent AI, in the name of efficiency, from becoming a source of technological pressure that controls teachers, replaces professional judgment, or redirects teaching around system requirements. Research on the datafication of higher education has similarly warned that data systems may reshape pedagogical discretion and narrow the space of professional judgment (Williamson et al., 2020). Work on AI ethics in education also argues that fairness, transparency, privacy, and accountability should be treated as preconditions for AI implementation, not as afterthoughts (Memarian and Doleck, 2023; Nguyen et al., 2023).

## 6 Conclusion

### 6.1 Summary of the study

This study drew on TAM, UTAUT, teacher professional agency, and embodied physical education to examine Chinese university physical education teachers' AI use intention profiles through Q methodology, identifying four distinct viewpoints (Davis, 1989; Venkatesh et al., 2003; Aartun et al., 2022; Priestley et al., 2026). The first profile positioned AI as a practical assistant for improving routine teaching efficiency. The second emphasized that AI use should remain within the embodied boundaries of physical education, where bodily demonstration, on-site judgment, and professional responsibility are central. The third profile-oriented AI toward research design, literature work, and academic writing, while the fourth foregrounded information overload, privacy risks, assessment fairness, and accountability pressure. The Q-sort results and post-sorting explanations together supported these findings, showing that Chinese university physical education teachers' AI use intention cannot be reduced to simple acceptance or rejection. Rather, it reflects differentiated professional positions formed around routine teaching tasks, embodied pedagogy, academic production, and risk governance. These findings align with recent work showing that AI is reshaping teacher development and digital competence in physical education (Wang and Wang, 2024), as well as research indicating that Chinese higher education-based physical education teacher educators face growing professional learning needs in research activities (Gong et al., 2024). Overall, the meaning of AI for university physical education teachers depends less on its general usefulness than on whether it can fit concrete work tasks, support their multiple academic roles, respect the embodied nature of physical education, and operate within controllable boundaries of privacy, fairness, and accountability.

### 6.2 Theoretical and practical implications

This study makes three theoretical contributions. First, it extends AI adoption research by moving beyond a single acceptance logic and showing that Chinese university physical education teachers' AI use intention is organized through multiple professional profiles. Rather than treating intention as a linear movement from acceptance to rejection, this study identified four distinct viewpoints, namely efficiency-oriented, embodied-boundary, research-support, and risk-burden profiles. These profiles show that AI use intention is shaped by work tasks, professional judgment, and institutional conditions. Second, the study advances a subject-specific understanding of AI in physical education. Because physical education places bodily demonstration, movement observation, immediate correction, and safety management at the center of teaching, AI-generated content or automated feedback cannot be directly transferred into classroom practice. It must be translated through sport-specific tasks, motor skill structures, student bodily differences, and safety requirements. Third, the study proposes a professional work adoption perspective. The four profiles indicate that AI use among university physical education teachers is not limited to classroom technology adoption. It is also embedded in research, grant writing, administrative documentation, teaching preparation, and risk governance. This perspective helps explain why AI may be accepted as an efficiency tool in one context, treated as a research assistant in another, and resisted when it creates additional responsibility or weakens professional control.

For practice, the findings offer four implications. First, universities and physical education departments should avoid uniform AI training. Support should be differentiated according to teachers' work tasks. Routine-work training may focus on physical fitness data processing, teaching information management, administrative writing, and feedback reports, while research-oriented support may focus on literature searching, grant preparation, English academic writing, and responsible AI use. Second, AI use in physical education should remain teacher-led. AI may support post-class video analysis, fitness feedback, tiered exercise suggestions, and teaching material preparation, but physical demonstration, real-time correction, load adjustment, student state recognition, and classroom safety should remain under teachers' final judgment. Third, universities should develop clear rules for AI use in physical education, especially for the collection, storage, anonymization, access, and interpretation of student movement data. AI-assisted evaluation should not be treated as equivalent to teachers' professional assessment. Fourth, administrators should prevent AI from becoming a new source of digital workload. If AI use leads to extra platform check-ins, documentation, reporting, or responsibility proof, it may shift from a support resource to a source of pressure. The priority should be explainable, controllable, and accountable AI practices that serve teaching quality, teacher development, and curriculum improvement.

### 6.3 Limitations and future research

Although this study identified meaningful AI use intention profiles among Chinese university physical education teachers, several research boundaries should be noted. First, the study was situated in Chinese higher education, where teacher evaluation, research expectations, physical fitness testing, curriculum administration, and institutional AI policies may shape teachers' views in context-specific ways. Future research could examine whether similar profiles emerge among university physical education teachers in different national, cultural, and institutional contexts. Second, Q methodology is designed to identify shared subjective viewpoints rather than estimate the population size of each profile. The four profiles should therefore be interpreted as theoretically meaningful viewpoint structures identified within this Q-sample, rather than as representative categories of all university physical education teachers. Future studies could combine Q methodology with large-scale surveys, latent profile analysis, structural equation modeling, or follow-up interviews to examine the distribution, antecedents, and outcomes of these profiles. Such work could also explore how different profiles are associated with teachers' actual AI use in teaching, research, assessment, and administrative work. In addition, because the Q-sorting task was conducted online, participants completed the sorting process without face-to-face facilitation. This format may have influenced their engagement with the task, their interpretation of some statements, or the depth of reflection involved in forced-distribution choices. Future research could combine online Q-sorting with follow-up interviews, or supervised sorting sessions to better capture how participants construct and explain their rankings. Third, the study used a cross-sectional design, which limits its ability to capture changes in teachers' AI use intention over time. Future longitudinal studies, repeated Q studies, classroom observations, and practice-based case studies could trace how these profiles evolve as teachers gain more experience with AI, and whether AI eventually reduces workload, supports professional development, or creates new responsibilities.

## Data Availability

The original contributions presented in the study are included in the article/supplementary material, further inquiries can be directed to the corresponding author.
