# Supplementary material for: Exploring university physical education teachers' artificial intelligence use intention profiles: a Q-methodology study
Source: Front Psychol. 2026 Jul 9;17:1895365. doi: 10.3389/fpsyg.2026.1895365 (PMC13392487; doi:10.3389/fpsyg.2026.1895365)
Supplement: Supplementary file 1 [file Supplementary_file_1.docx]

**Appendix 1. Complete Q-set Statements and Factor Array Scores**

| **Statements** | **F-1** | **F-2** | **F-3** | **F-4** |
| --- | --- | --- | --- | --- |
| 1. AI can improve my efficiency in physical education teaching work. | 5 | -1 | 3 | -1 |
| 2. AI tools that are easy to operate are more likely to be used continuously by me. | 4 | 0 | -1 | 0 |
| 3. I am willing to actively explore the application of AI in physical education teaching. | 1 | -3 | -1 | -2 |
| 4. Institutional support would increase my willingness to use AI. | 0 | -2 | 0 | -1 |
| 5. Colleagues’ successful experiences would encourage me to try using AI. | 2 | -2 | -1 | -2 |
| 6. Students’ acceptance of AI would influence my willingness to use it in class. | 2 | -2 | -1 | 0 |
| 7. Practical effectiveness is an important basis for my decision to use AI. | 3 | 2 | 2 | 0 |
| 8. I pay more attention to the practical value of AI in physical education teaching. | 3 | 1 | 1 | 0 |
| 9. I am confident that I can learn commonly used AI tools for physical education teaching. | 1 | -1 | 3 | -1 |
| 10. Systematic training would increase my confidence in using AI. | 1 | 0 | 2 | 0 |
| 11. The rapid updating of AI technology would bring me adaptation pressure. | 0 | 0 | 0 | 3 |
| 12. I can judge whether AI tools are suitable for physical education teaching contexts. | 0 | 3 | -1 | 1 |
| 13. I can transform AI-generated content into physical education teaching materials. | -1 | -3 | -2 | -3 |
| 14. I can identify problems in AI-generated content. | -1 | 3 | 2 | 1 |
| 15. Improving AI competence is an important part of my professional development. | 2 | 0 | 3 | -2 |
| 16. The key for physical education teachers to adapt to AI lies in professional judgment. | -1 | 3 | 1 | 1 |
| 17. AI can help me design personalized physical education teaching plans. | 0 | 0 | 0 | -5 |
| 18. AI can provide differentiated exercise suggestions based on students’ physical fitness differences. | 1 | 2 | -4 | -5 |
| 19. AI can assist in analyzing students’ movement techniques. | -2 | -4 | -4 | -4 |
| 20. AI can improve the efficiency of organizing physical fitness test data. | 4 | 2 | -3 | -1 |
| 21. AI can help me promptly understand changes in students’ sports performance. | -1 | -5 | -2 | -2 |
| 22. AI is more suitable for assisting physical education theory classes. | -2 | 2 | -3 | -4 |
| 23. Physical skill teaching relies more on teachers’ on-site judgment. | -2 | 4 | 1 | 2 |
| 24. The complex situations of physical education classes would limit the effectiveness of AI application. | 0 | 5 | 1 | 2 |
| 25. AI is more suitable as an auxiliary tool in physical education teaching. | 5 | 5 | 0 | 2 |
| 26. AI can improve my efficiency in literature searching. | 3 | -3 | 4 | -1 |
| 27. AI can help me improve the expression of English academic papers. | 2 | -3 | 3 | -4 |
| 28. AI can assist me in conducting research design. | -3 | -4 | 5 | -3 |
| 29. AI can improve the writing efficiency of grant proposal materials. | 3 | -2 | 4 | -3 |
| 30. AI can reduce the time spent writing routine administrative materials. | 4 | -1 | 2 | 1 |
| 31. I am more willing to use AI first in research work. | -3 | -5 | 5 | -3 |
| 32. Teachers’ research judgment should guide the use of AI-generated content. | -1 | 0 | 4 | 0 |
| 33. Deep involvement of AI in paper writing would bring academic norm pressure. | -5 | -4 | 0 | 4 |
| 34. AI application would change the professional role of physical education teachers. | 0 | 1 | -2 | 3 |
| 35. Physical education teachers’ bodily demonstration has unique value. | -2 | 4 | 1 | 2 |
| 36. AI-based evaluation would influence the professional authority of physical education teachers. | -5 | 1 | -5 | 1 |
| 37. AI application would increase technology-related work requirements for physical education teachers. | -4 | 1 | -3 | 4 |
| 38. Learning AI would increase my additional workload. | -4 | -1 | -4 | 3 |
| 39. Too many AI tools would cause information overload. | -4 | -1 | -3 | 5 |
| 40. AI processing of students’ sports data would bring privacy risks. | -3 | 4 | -2 | 4 |
| 41. AI evaluation of students’ sports performance would bring fairness issues. | -3 | 3 | -5 | 5 |
| 42. The responsibility boundaries of AI use would affect my confidence in using it. | 1 | 1 | 0 | 3 |
